# Epithelial-mesenchymal transition and immunosuppression: Two sides of the same coin

**DOI:** 10.1007/s00018-026-06240-y

**Published:** 2026-06-06

**Authors:** Brian Feng, Anushka Dongre

**Affiliations:** Department of Biomedical and Translational Sciences, College of Veterinary Medicine, Cornell University, Ithaca, NY 14853 USA

**Keywords:** Epithelial-mesenchymal transition, Immmunosuppression, Immunotherapy, Tumor microenvironment, Metastasis

## Abstract

Epithelial-mesenchymal transition has long been studied as a physiologic process that is critical for embryonic development, wound healing, and cancer progression. Activation of the EMT program confers on cancer cells many cellular traits associated with high-grade malignancies. These include invasion, motility, the acquisition of stem cell-like features, and resistance to multiple therapies. An emerging frontier of the EMT program, is its ability to regulate the activity of immune cells within tumors and drive resistance to immune checkpoint blockade therapies. In this review, we provide an overview of the mechanistic underpinnings of EMT-driven immunosuppression on specific subsets of innate and adaptive immune cells present within primary tumors. We also provide an insight into the role of various EMT-transcription factors in regulating the expression of cancer cell-intrinsic immunosuppressive and immunoevasive factors. Additionally, we review the consequences of EMT-driven immunosuppression on distinct steps of the invasion-metastasis cascade. Finally, we summarize studies that document EMT-driven immunosuppression in multiple cancer types.

## Introduction

Epithelial-mesenchymal transition (EMT) is a biological program in which epithelial cells acquire mesenchymal features [1–4]. This central process drives early development, wound healing, fibrosis, and cancer progression [5, 6]. EMT is characterized by the upregulation of transcription factors including SNAI1 (SNAIL), SNAI2 (SLUG), ZEB1, and TWIST1 [7–10]. Cells undergoing a complete EMT lose the expression of epithelial markers like E-cadherin and gain the expression of mesenchymal markers like N-cadherin, Fibronectin, and Vimentin. However, the transition from an epithelial to a mesenchymal state is rarely unidirectional or binary [11–13]. Instead, activation of the EMT program is a plastic process in which interconversion between intermediate states is observed, often referred to as epithelial-mesenchymal plasticity (EMP) [14]. Malignant EMT programs can lead to a partial epithelial-mesenchymal or quasi-mesenchymal (qM) state [15–18]. Cells in intermediate EMT states may co-express epithelial markers including E-cadherin and Cytokeratins, alongside mesenchymal markers such as Vimentin. Alternatively, these cells may reside in a partial EMT state characterized by the downregulation of epithelial markers without a corresponding upregulation of mesenchymal markers [2]. Many studies have demonstrated the role of EMT in cancer immunosuppression and immunotherapy resistance [19–23]. However, the precise effects of EMT on immune cells and cancer immunotherapies is an evolving field and requires further investigation to improve diagnostic potential and inform treatment strategies.

The tumor microenvironment (TME) is a unique ecosystem made up of immune cells, fibroblasts, blood vessels, and the extracellular matrix [24]. Structurally distinct from normal tissue, the TME is metabolically and immunologically dysregulated. During cancer progression, EMT drives immunosuppression in the TME by increasing the expression of EMT transcription factors (EMT-TFs), promoting the secretion of pro-tumor paracrine factors and cytokines, suppressing innate and adaptive immune cells, reducing antigen presentation, and upregulating immune checkpoints [19–21, 23, 25–27]. In addition, EMT promotes the acquisition of stem cell-like properties of cancer cells and facilitates metastases to distant sites, which is the leading cause of death in many cancers [28–30]. These effects can render immunotherapies ineffective. The heterogeneity of EMT states as well as differences in immunotherapeutic responses in various cancer types underscores the need to investigate cancer type-specific effects of EMT on immunoevasion.

Research on EMT and cancer immunosuppression holds tremendous potential for improving cancer prognoses. However, the precise mechanisms by which EMT contributes to immunosuppression, and which therapeutic targets can be exploited requires further study. In this review, we provide a comprehensive overview of (i) how EMT affects immune cells in the TME, (ii) the impact of EMT on the efficacy of immunotherapies, (iii) differences in immune responses in the primary tumor vs metastatic sites, and (iv) the immunosuppressive effects of EMT in different cancer types. Future studies dissecting the immunosuppressive effects of cancer EMT programs will provide critical insights into improving therapeutic strategies.

### EMT transcription factors and immunosuppression

**Fig. 1:**
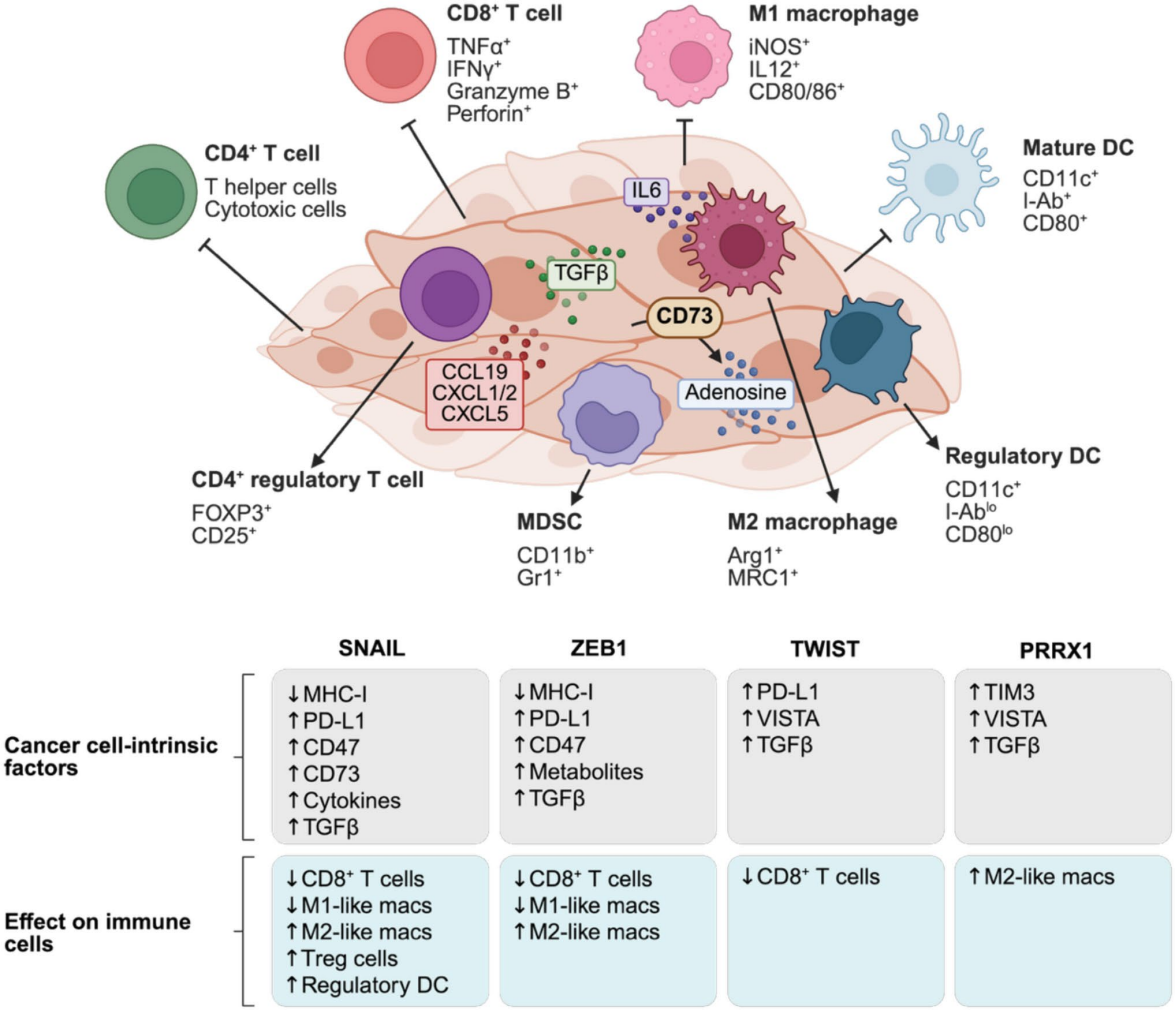
Mechanisms by which mesenchymal cancer cells induce immunosuppression in the primary tumor microenvironment. EMT-like mesenchymal cancer cells assemble an immunosuppressive tumor microenvironment by expressing multiple cancer cell-intrinsic immunosuppressive factors. These tumors contain immunosuppressive regulatory T cells, M2-like pro-tumor macrophages, myeloid derived suppressor cells (MDSC) and regulatory dendritic cells (DC). They exclude M1-like anti-tumor macrophages, effector CD4^+^ and CD8^+^ T cells, and conventional dendritic cells (DC). Arrowheads indicate cell types induced by mesenchymal tumor cells. Blunt-headed arrows indicate cell types inhibited by mesenchymal tumor cells. Boxes at the bottom represent specific effects of each EMT-TF on (i) the identity of cancer cell-intrinsic markers of immunoevasion and immunosuppression and (ii) the effect on immune cells within the primary tumor

EMT transcription factors (EMT-TFs) are key regulators of signaling pathways and cellular processes that drive cancer cells towards a mesenchymal state. EMT-TFs control the expression of cancer cell-intrinsic immunosuppressive factors, enabling cancers cells to evade anti-tumor immune responses. Here, we discuss how EMT-TFs regulate immune-related genes, and how these changes subsequently alter the composition of the TME (Fig. 1).

### SNAIL

SNAIL is a zinc-finger transcription factor that is required for early embryogenesis and EMT in several cancer types [7, 8, 31]. While the role of SNAIL in driving EMT and metastasis is well established, many studies have also demonstrated its ability to promote immunosuppression in the TME [19, 32–34]. These mechanisms include inhibiting antitumor T cells, inducing suppressive immune cells, decreasing antigen presentation, increasing cancer-cell intrinsic immunosuppressive factors, and reducing susceptibility of tumors to macrophage killing. SNAIL plays a key role in promoting immunosuppression by inhibiting the infiltration and cytotoxic function of CD8^+^ T cells [19–21]. Using ATAC seq data, Kim and colleagues demonstrated that SNAIL and ZEB1 overexpression resulted in reduced accessibility to IRF6 in tumor cells [35]. When IRF6 was restored, it also restored CD8^+^ T cell killing by TNFα secretion. SNAIL upregulation also results in decreased MHC-I expression on cancer cells, critical for presenting tumor antigens to cytotoxic CD8^+^ T cells [21, 32]. In prostate cancer, elevated TGFβ and Epidermal growth factor (EGF) increases SNAIL expression on cancer cells, which may target NF-κB signaling, resulting in the downregulation of MHC-I [32]. Similarly, a study comparing quasi-mesenchymal SNAIL^hi^ and epithelial SNAIL^lo^ PyMT murine mammary tumors revealed that SNAIL^lo^ tumors exhibited high MHC-I expression while SNAIL^hi^ quasi-mesenchymal tumors displayed low MHC-I expression [21].

In addition to regulating immune cells and cancer-cell intrinsic antigen presentation, SNAIL can also induce immunosuppression by transcriptionally upregulating immunomodulatory genes and immune checkpoint molecules on cancer cells. Expression of these molecules allow cancer cells to efficiently evade immune attack. One study found that SNAIL induction resulted in CD73 upregulation via direct promoter-proximal binding. This increase in CD73 resulted in immunosuppression [36]. Dongre and colleagues reported similar findings using ChIP-seq data in quasi-mesenchymal cells [15]. They found that SNAIL bound to the promoter regions of immunosuppressive factors NT5E (CD73), CSF1, and SPP1. In PyMT mammary carcinoma cells undergoing EMT, upregulation of SNAIL coincides with an increase in PD-L1 expression [21]. Increased PD-L1 expression can directly inhibit susceptibility to CD8^+^ T cell killing by promoting T cell exhaustion. Analogous to this observation, SNAIL can upregulate macrophage immune checkpoints to inhibit killing by intratumoral macrophages. Noman and colleagues observed increased expression of the CD47 macrophage “don’t eat me” signal in MCF7 human breast cancer cells in response to SNAIL upregulation via direct binding of SNAIL to E-boxes in the promoter of CD47 [37]. Finally, studies in PyMT murine mammary carcinoma and human head and neck cancer models revealed that SNAIL can influence macrophage state in the tumor by facilitating EMT-mediated M2-like macrophage polarization characterized by expression of Arginase-1 and MRC1 [21, 38]. In addition, Brenot and colleagues determined that SNAIL inhibits GM-CSF, a potent stimulus of M1-like macrophage polarization [33]. Collectively, these seminal studies establish SNAIL as a multi-pronged driver of immunosuppression and evasion in cancer.

### ZEB1

Zinc finger E-box binding homeobox 1 (ZEB1) is a TF that drives EMT and invasion in several cancer types including non-small cell lung carcinoma (NSCLC) [39], colorectal cancer [40, 41], pancreatic cancer [42] and breast cancer [41]. ZEB1 drives EMT by increasing expression of migration and invasion factors including LOXL2, repressing the expression of cell polarity factors such as Lethal giant larvae homolog 2 (LGL2) and epithelial factors such as epithelial cadherin (E-cadherin) [9, 40, 41]. ZEB1 is also a potent mediator of immunosuppression in the TME. ZEB1 represses members of the miR-200 family, which normally inhibits the expression of PD-L1 on cancer cells. As a result, PD-L1 expression increases, leading to the suppression of CD8^+^ T cell antitumor activity and metastasis [43–45]. High PD-L1 expression on cancer cells presents a significant challenge for patients and has been linked to poor prognosis in several cancer types [46–50]. In addition to inhibiting adaptive immune responses, ZEB1 upregulation can also impair innate immune cells. In a study that evaluated EMT reversal in breast cancer, restoration of miR-200c in Met-1 MMTV-PyMT and human TNBC cell lines resulted in a more epithelial-like state and increased Nitric oxide synthase 2 (Nos2)-expressing M1-like macrophages [51]. Similar to the activity of SNAIL, ZEB1 can increase CD47 expression on cancer cells via binding to CD47 promoter E-boxes, resulting in impaired macrophage phagocytosis, further promoting immunoevasion [37, 52]. These findings reveal the importance of ZEB1 as a mediator of both innate and adaptive immunosuppression and metastasis.

### TWIST1

TWIST1 is a transcription factor that drives EMT and mesoderm development of the cranial neural tube during embryogenesis [53, 54]. In cancers, its expression is primarily associated with invasion and metastasis, and only a few examples of immunosuppression [55]. Xu and colleagues demonstrated that TWIST1 expression in PyMT mammary luminal epithelial primary tumors causes a partial EMT, resulting in intravasation and lung metastasis [56]. Moreover, knocking out TWIST1 silenced the expression of other EMT transcription factors such as ZEB2, SNAIL, and SLUG, and prevented intravasation and lung metastasis [56]. TWIST1 is also known to activate immune checkpoint proteins. For instance, TWIST1 is a transcriptional activator of PD-L1, which directly inhibits the activity of CD8^+^ T cells. Knockdown of TWIST1 in MCF7 cells resulted in the reinvigoration of CD8^+^ T cells in breast tumors [57]. In pancreatic cancer, TWIST1 is also known to positively regulate VISTA, a B7 family immune checkpoint protein, leading to increased migration and progression [58]. Together, these studies highlight the importance of TWIST1 as a driver of invasion and metastasis as well as T cell suppression.

### PRRX1

Another driver of EMT is the homeobox factor PRRX1. Paradoxically, while PRRX1 promotes EMT, invasion, and migration in the primary tumor, its loss is required for the acquisition of stem-like features and metastatic colonization at secondary sites *in vivo* [59]. When PRRX1 is lost from mesenchymal cancer cells, a complete mesenchymal-epithelial transition (MET) occurs. Epithelial cells that result from this MET are characterized by a CD44^+^CD24^−^ stem cell-like expression pattern and greater tumor initiating ability [59]. In contrast to other EMT-TFs described in this review, low PRRX1 expression is correlated with lower relapse-free survival in breast adenocarcinoma and lung squamous cell carcinoma [59–61]. Another study evaluated whether a mesenchymal phenotype could be identified within patient metastatic colorectal cancer data [62]. They identified a PRRX1 gene signature associated with a mesenchymal and immune phenotype characterized by an abundance of CD4^+^ and CD8^+^ T cells, dendritic cells, macrophages, neutrophils, and immune activation characterized by IFNγ signaling. However, they also predicted this signature to be enriched for TGFβ-driven immunosuppression as evidenced by differentially expressed genes [62]. PRRX1 was also positively correlated with CD163 expression, a marker of M2-like immunosuppressive macrophages. Interestingly, PRRX1 high tumors had greater expression of TIM-3 and VISTA immune checkpoints, indicating a suppressive effect on T cells [62]. Clinically, the authors observed no significant differences between PRRX1^hi^ tumors and the remaining cohort in overall survival. Together, these studies demonstrate that in contrast to other EMT-TFs, PRRX1 represents a unique case of decoupling primary tumor EMT and disease outcome. Moreover, PRRX1 may act as a unique biomarker for predicting immunotherapy efficacy due to its association with an immune cell-enriched phenotype.

### EMT and immune cells in the tumor microenvironment

The tumor microenvironment (TME) is a complex system composed of cancer cells, cancer-associated fibroblasts (CAFs), immune cells, secreted factors, and vasculature [63, 64]. During cancer progression, the transition to a hybrid or mesenchymal state creates an immunosuppressive environment that enables the tumor to proliferate while evading immune attack. These effects include impairing T cell infiltration, promoting T cell exhaustion, upregulating immunosuppressive regulatory T cells (Tregs), and inducing immunosuppressive M2-like macrophage polarization. Here we discuss the immune cell-specific suppressive effects of EMT. Characterizing how immune cells change during EMT will offer new insights into therapeutic strategies to target cancer cell plasticity and immunosuppressive features of the TME (Table 1).

**Table 1:** EMT drives immunosuppression and resistance to immunotherapies in multiple cancer types

Cancer type	Immune cell	Status in EMT-like tumors	Response to immunotherapy
Breast	CD8^+^ T cells CD4^+^ T cells Macrophages Dendritic cells Cancer cell- intrinsic factors	Increased dysfunctional CD8^+^ T cells [15, 21, 78], Reduced or excluded effector CD8^+^ T cells [15, 21, 78], Attenuation of immunologic synapse [20], Increased Tregs [21, 78] Increased M2-like macrophages [21, 34, 51, 78, 91]. Reduced M1-like macrophages [21, 34, 51, 78] Reduced cDC1 [21] Reduced antigen presentation [20, 21], Increased PD-L1 [21, 44], Increased CD47 [44], Increased adenosinergic signaling CD73 [21] CD38 [78], Increased cytokines and chemo attractants [15, 91], Increased metabolites [51]	Resistance to immune checkpoint blockade therapy anti-CTLA4 [15], anti-PD1 [15], anti-PD-L1 [78], anti-CTLA4 in combination with anti-PD1 [91] in murine models
Lung	CD8^+^ T cells CD4^+^ T cells Cancer cell- intrinsic factors	Increased dysfunctional CD8^+^ T cells [43, 132]. Reduced infiltration into the tumor core [132] Increased Tregs [133] Reduced antigen presentation, Increased PD-L1 and immune checkpoints [22, 43], Increased CD70 [132]	Predictive of poor response to immune checkpoint blockade therapy (anti-PD-L1) in patients [133]
Melanoma	CD8^+^ T cells CD4^+^ T cells Dendritic cells Cancer cell- intrinsic factors	Impaired CD8^+^ T cell recruitment [19, 77] Increased Tregs [19, 77] Increased Tolerogenic DCs [19] Increased PD-L1 [134], Decreased T cell chemoattractants [77]	Resistance to immune checkpoint blockade therapy anti-CTLA4 [19] and anti-PD1 [77] in murine models
Pancreatic	CD8^+^ T cells Macrophages Cancer cell- intrinsic factors	Fewer CD8^+^ T cells [135]. Active CD8^+^ T cells but cancer cells resistant to killing [35] Increased M2-like macrophages [136] Increased immunomodulatory factors [136]	Acquired resistance to chemo-immunotherapy (gemcitabine, nab-paclitaxel, anti-CTLA4, anti-PD1) in murine models [35]
HNSCC and OSCC	CD8^+^ T cells Macrophages Cancer cell- intrinsic factors	Increased dysfunctional CD8^+^ T cells [137] Increased M2-like macrophages [89] Increased immune checkpoint molecules, reduced antigen processing and presentation, increased immunosuppressive factors [137–139]	NA
Ovarian	CD8^+^ T cells CD4^+^ T cells Macrophages NK cells MDSCs	Increased dysfunctional CD8^+^ T cells [79] Resting memory T cells [140] Increased M2-like macrophages [140, 141] Resting NK cells [140] Increased MDSCs [142]	NA
Colon and Colorectal	CD8^+^ T cells CD4^+^ T cells NK cells Dendritic cells Macrophages Cancer cell- intrinsic factors	Increased dysfunctional CD8^+^ T cells [143] Increased Tregs, Th2 cells [143, 144] Reduced NK cells [144] Increased dysfunctional dendritic cells [144] Increased M2-like immunosuppressive macrophages [143–145] Increased proinflammatory and immunosuppressive factors, Increased expression of immune checkpoints [143, 144]	NA
Bladder	Cancer cell- intrinsic factors	Increased PD-L1 [146]	NA

*HNSCC* Head and neck squamous cell carcinoma; *OSCC* Oral squamous cell carcinoma; *MDSCs* Myeloid derived suppressor cells; *DCs* Dendritic cells; *cDC1* Conventional dendritic cell type I; *Tregs* T-regulatory cells

### CD8^+^ T cells

Immunosuppression in the TME can inhibit T cell function and infiltration into the tumor core. Under normal conditions, T cell activation consists of three signals: i) when a T cell encounters a tumor antigen presented by major histocompatibility complex (MHC) molecules on an antigen presenting cell, its T cell receptor engages it and transmits downstream activation signals. ii) A second signal takes place between a costimulatory receptor on the T cell such as CD28 and its ligand CD80/86, required for proliferation. iii) A third signal involves the milieu of cytokines encountered by the T cell, which instructs T cell differentiation into subsets with various functions [65]. CD8^+^ T cells are widely regarded as cytotoxic effector T cells that recognize MHC-I-restricted antigens and secrete effector cytokines and lytic granules to eliminate cancer cells. In contrast, and with the exception of Tregs, CD4^+^ T cells are considered helper T cells which recognize MHC-II-restricted antigens and stimulate the activity of other immune cells including CD8^+^ T cells [66–68]. More recently, several studies have revealed the cytotoxic potential of CD4^+^ T cells in cancer [69–73].

CD8^+^ T cell infiltration in the tumor is a key predictor of disease outcome and response to immunotherapy. When CD8^+^ T cell infiltration is impaired, tumors become resistant to killing, prone to metastasis, and unresponsive to immunotherapies [74, 75]. In a study that evaluated the effects of EMT on cytotoxic lymphocyte-mediated lysis in MCF7 human breast cancer cells, cell lines induced to undergo EMT exhibited significant impairment of cytotoxic lymphocyte killing due to the induction of autophagy in cancer cells [20]. Similarly, using a murine model of spontaneous mammary tumor formation (MMTV-PyMT), another study revealed that quasi-mesenchymal tumors exhibited reduced infiltration by CD8^+^ T cells and iNOS^+^IL12^+^ M1-like macrophages accompanied by an increase in CD4^+^CD25^+^FOXP3^+^ immunosuppressive Tregs compared to epithelial tumors [21]. Another study demonstrated a link between EMT in melanoma cells and increasing cancer cell invasion and immunosuppression characterized by reduced CD8^+^ T cells [19].

Several other tumor-intrinsic factors have been implicated in restricting CD8^+^ T cell infiltration. In pancreatic cancer, expression of FAM83H, an oncogene involved in β-catenin stabilization, was correlated with both EMT and reduced CD8^+^ T cell infiltration [76]. Plaschka and colleagues demonstrated that ZEB1 directly suppresses the secretion of CXCL10, a CD8^+^ T cell chemoattractant [77]. Adenosinergic signaling is also a key EMT-associated pathway that inhibits immune cells in the TME, including CD8^+^ T cells. In qM PyMT mammary tumors, expression of CD73, an ectoenzyme that converts extracellular AMP to adenosine, is associated with CD8^+^ T cell exclusion and reduced T cell effector function [15]. Similarly, Visal and colleagues demonstrated that CD38, an enzyme upstream of CD73, was a crucial driver of the hybrid E/M state in TNBC [78]. When CD38 was targeted, cytotoxic CD8^+^ T cells in the tumor increased [78].

In addition to reducing infiltration, EMT-induced immune suppression can also promote the exhaustion of CD8^+^ T cells in the tumor. One main driver of this exhaustion is the expression of immune checkpoint ligands such as PD-L1 on tumor cells. For instance, tumors undergoing EMT upregulate PD-L1 expression, leading to CD8^+^ T cell exhaustion [43, 44, 57]. Combined with chronic antigen stimulation, which increases PD1 expression on CD8^+^ T cells, T cell exhaustion becomes more pronounced. In ovarian cancer, EMT is associated with increased expression of LGALS3 which can bind to the LAG3 receptor on CD8^+^ T cells, further contributing to their exhaustion [79].

Not only can EMT in tumor cells induce changes in T cells of the surrounding environment, but it can also promote tumor-intrinsic resistance to immune attack. For instance, Kim and colleagues demonstrated in a mouse model of PDAC that EMT renders OVA-expressing tumor cells unresponsive to the proapoptotic effects of TNFα derived from OT-I CD8^+^ T cells [35].

### Tregs

CD4^+^ T cells are typically described as helper T cells that stimulate the activity of other immune cells. CD4^+^ T cells recognize peptides presented by MHC-II molecules on the surface of antigen-presenting cells (APCs) and may differentiate into several helper or effector subsets. CD4^+^ T cells can also produce IFNγ, which stimulates myeloid cells to secrete tumoricidal nitric oxide [80]. More recent studies have revealed the direct cytotoxic capabilities of CD4^+^ T cells. For instance, CD4^+^ T cells can directly recognize peptides presented by MHC-II on cancer cells and secrete effector cytokines and granules such as IFNγ and TNFα [81]. CD4^+^ T cells have also been shown to produce Perforin and Granzyme B similarly to cytotoxic CD8^+^ T cells during influenza viral infection and in an engineered melanoma model [82, 83]. One method of indirect regulation is dendritic cell licensing where CD4^+^ T cells stimulate dendritic cells to promote cytotoxic CD8^+^ T cell function [71, 81]. CD4^+^ T cells can also induce tumor cell apoptosis through death-receptor ligand-mediated pathways such as FASL-FAS and TRAIL [84]. In addition to its anti-tumor functions, CD4^+^ T cell subsets including FOXP3^+^CD25^+^ CD4^+^ T cells can have immunosuppressive effects in the TME.

Many studies across different cancer types have demonstrated the ability of EMT to induce immunosuppressive Tregs in the TME. For instance, Kudo-Saito and colleagues revealed that melanoma tumors undergoing EMT contained elevated numbers of Tregs [19]. Furthermore, *in vitro* studies using human CCA Cholangiocarcinoma cell line-peripheral blood mononuclear cell (PBMC) cocultures demonstrated that a SNAIL knockdown-induced EMT resulted in a decrease in immunosuppressive CD4^+^ FOXP3^+^ Treg induction [33]. In melanoma cells, expression of ZEB1 led to an increase in Treg proportions [77]. These studies highlight the direct link between EMT TF upregulation and a shift towards an immunosuppressive Treg-dominant TME.

Additional studies in CD73-expressing murine mesenchymal breast carcinoma revealed a TME characterized by an abundance of CD25^+^FOXP3^+^ Tregs and fewer cytotoxic CD8^+^ T cells [21]. Furthermore, Visal and colleagues demonstrated in CD38-expressing hybrid epithelial-mesenchymal TNBC tumors, that CD38 knockdown leads to increased CD4^+^ T cells and decreased FOXP3^+^ Tregs [78]. These findings are particularly notable as they highlight the immunomodulatory effects of cancer cell-intrinsic CD38 and CD73 upregulation, both ectoenzymes that cooperate to generate extracellular adenosine, a well-documented driver of Treg expansion. Collectively, these studies suggest that EMT not only facilitates the exclusion and dysfunction of effector T cells, but it also promotes the differentiation and expansion of immunosuppressive cells including Tregs. This further emphasizes the tumor-extrinsic immune remodeling effects of EMT.

### Macrophages

Macrophages can adopt either pro- or anti-tumor functions depending on which state they reside in. Pro-inflammatory M1-like macrophages are induced by IFNγ and Th1 cytokines and characterized by expression of nitric oxide synthase 2 (NOS2), CD80/86 costimulatory molecules, MHC-II, and suppressor of cytokine signaling (SOCS)1 [85]. These anti-tumor macrophages can secrete nitric oxide, engage in antibody-dependent cell-mediated cytotoxicity (ADCC) whereby they recognize antibody coated targets via their Fc receptors and engulf them, phagocytose cancer cells, and activate T cells and NK cells [86–88]. Anti-inflammatory M2-like macrophages are induced by IL-10, IL-4 and IL-13 and express Arginase-1 (ARG1) [85]. These pro-tumor macrophages promote tumorigenesis through angiogenesis, immunomodulation of T cell and NK cells, support EMT, and facilitate intravasation and metastasis. Despite the existence of M1-like and M2-like macrophages, macrophage heterogeneity is far more complex and encompasses a spectrum of states and functions in the tumor microenvironment [86].

While many studies have demonstrated the effects of M1-like and M2-like macrophages on immunosuppression in the TME, here we discuss the role of EMT-driven immunosuppression in reprogramming macrophages. SNAIL is a key regulator of tumor associated macrophage polarization. In a comparison of 4T1 murine breast cancer cells either overexpressing SNAIL or a control vector, 4T1-SNAIL cells contained more M2-like macrophages [89]. Furthermore, Dongre and colleagues demonstrated that epithelial PyMT tumors contained M1-like anti-tumor macrophages while qM tumors contained more M2-like pro-tumor macrophages [21]. These studies suggest that macrophage polarization is tightly linked to EMT state. Moreover, Williams and colleagues demonstrated that epithelial-like breast tumors contained more Nitric oxide synthase 2 (Nos2)-expressing M1-like macrophages [51]. Visal and colleagues showed that in addition to CD8^+^ T cells, CD38 suppression also resulted in an increase in M1-like iNOS^+^MHC-II^+^ macrophages [78]. As discussed with the induction of Tregs, these findings suggest that adenosinergic signaling is a broad inducer of suppressive immune cell states. Studies of metastatic breast carcinoma revealed that regions of more mesenchymal cells in the tumor contained more M2-like polarized macrophages than non-mesenchymal regions [90]. *In vitro* studies demonstrated that M2 macrophage polarization in this context was facilitated by CSF1. Similar to the mechanism of T cell inhibition, mesenchymal cancer cells promote the expression of the macrophage immune checkpoint protein CD47, inhibiting phagocytosis [37, 52].

### Other myeloid cells

While EMT can promote immunosuppression in the TME, it can also be driven by immune cells themselves. Several studies have demonstrated the role of myeloid cells, specifically tumor-associated macrophages (TAMs) and myeloid-derived suppressor cells (MDSCs), in driving and sustaining EMT during cancer progression. Here we focus on the effects of EMT-induced immunosuppression on myeloid cells including neutrophils, MDSCs, and dendritic cells in the TME. Using BALB/c and C57BL/6 murine mammary tumor models to study EMT and immune infiltration, Kim and colleagues identified both a macrophage-enriched subtype (MES) and a neutrophil enriched subtype (NES) [91]. The NES – MES classification strongly correlated with the Epithelial – Mesenchymal spectrum [91]. The authors also found that the gene encoding tumor necrosis factor-inducible gene 6 (TSG6), which binds to CXCL1/2, was expressed in three out of four MES tumors, thereby blocking neutrophil migration. In line with this evidence, miR-200, a negative regulator of ZEB1, was highly expressed in epithelial NES tumors. miR-200 overexpression in MES tumors coincided with increased CXCL1 neutrophil chemoattractant and less TSG6 expression. They also determined that CCR2 was required for tumor-infiltrating macrophage recruitment in MES but not in NES tumors. Tumor-infiltrating neutrophils in the NES subtypes were primarily MDSCs and had an immunosuppressive effect. Interestingly, MES tumors were more likely to respond to combinations of anti-CTLA4 and anti-PD1 treatment while NES tumors were refractory to this immune checkpoint blockade combination. These results demonstrated that myeloid cell identity and immunosuppressive state play a large role in dictating responses to immune checkpoint blockade therapies. EMT can also increase suppressive myeloid cells while reducing anti-tumor myeloid cells. For instance, mesenchymal tumors exhibited reduced infiltration by CD8^+^ T cells, FOXP3^lo^ CD4^+^ T cells, and I-A^b+^CD11c^+^ MHC-II antigen-presenting dendritic cells (DCs). Instead, these tumors contained dysregulated dendritic cells with lower costimulatory molecule expression and immunosuppressive naturally occurring CD4^+^ Foxp3^+^ Tregs [19]. Additional studies have shown that CCL2 and lipocalin-2 promote an EMT resulting in the generation of immunoregulatory DCs with decreased HLA-DR and increased PD-L1 in human PBMCs [92]. Finally, adenosinergic signaling in quasi-mesenchymal PyMT tumors results in increased suppressive DCs [15].

### Cytokines

The aggressive nature of mesenchymal tumors is partly attributable to their ability to secrete suppressive factors to modulate the activity of immune cells in the TME. TGFβ is a key cytokine secreted by a variety of cells including mesenchymal tumor cells, stromal cells, and immune cells. TGFβ has broad immune suppressive effects including the generation of suppressive Tregs, downregulation of perforin, granzyme B, and IFN-γ in cytotoxic T cells, induction of M2 macrophage polarization, inhibition of NK cell effector function, restriction of dendritic cell migration, and promotion of MDSC expansion [93–95]. Paracrine signaling via CD73-derived adenosine signaling similarly reduces the antitumor activity of immune cells. Other cytokines also play an important role in modulating immune cell function. In TNBC, MCT-1 is a marker of poor prognosis and is known to stimulate EMT. MCT-1 stimulates the secretion of IL6, which causes immunosuppressive M2-like macrophage polarization [96]. Chemoattractants such as CCL19, CXCL1, CXCL2, and CXCL5 recruit Myeloid-derived Suppressor Cells (MDSCs) which drives EMT and creates a positive feedback loop to recruit additional MDSCs, as demonstrated by Toh and colleagues in the spontaneous melanoma RETAAD model [97].

### EMT-induced immunosuppression and metastasis

An important consequence of EMT-induced immunosuppression is refractory responses to immune checkpoint inhibitors in multiple cancer types (Table 1). While immune checkpoint inhibitors have demonstrated tremendous clinical success, only a subset of tumors derive benefit, notably melanomas and non-small cell lung cancers [98–101]. Moreover, heterogeneous responses to immune checkpoint inhibitors necessitates the need for identifying predictive criteria that clearly separate responders from non-responders. The EMT program is emerging as one such parameter [102]. Dongre and colleagues demonstrated that quasi-mesenchymal breast tumors are resistant to anti-CTLA4 immune checkpoint blockade therapy, even when present at minority populations within primary tumors [21]. In the context of preclinical murine models of breast tumors, this resistance is driven by members of the adenosinergic signaling pathway as intercepting this pathway can enhance susceptibility of partial or quasi-mesenchymal tumors to immune checkpoint inhibitors [15, 78]. Similarly, preclinical murine models of melanoma that are either SNAIL or ZEB driven, demonstrate intrinsic resistance to checkpoint inhibition [19, 77]. Additionally, acquired resistance to combinations of chemo-immunotherapy has been observed in preclinical murine models of pancreatic cancer [35]. Finally, patient samples that display EMT signatures are also associated with immunosuppressive cells and elevated expression of immune checkpoint molecules (Table 1). The importance of EMT-driven resistance to checkpoint inhibitors is compounded by the fact that the EMT program also leads to metastatic colonization. Thus, strategies to target the EMT program hold the potential to concomitantly attenuate both immunosuppression and metastasis, thereby increasing the susceptibility of both primary tumors and distant metastasis to immune checkpoint blockade therapies. It is therefore pertinent to understand the mechanisms of EMT-induced immunosuppression during metastasis, in addition to those operating within quasi-mesenchymal primary tumors.

**Fig. 2:**
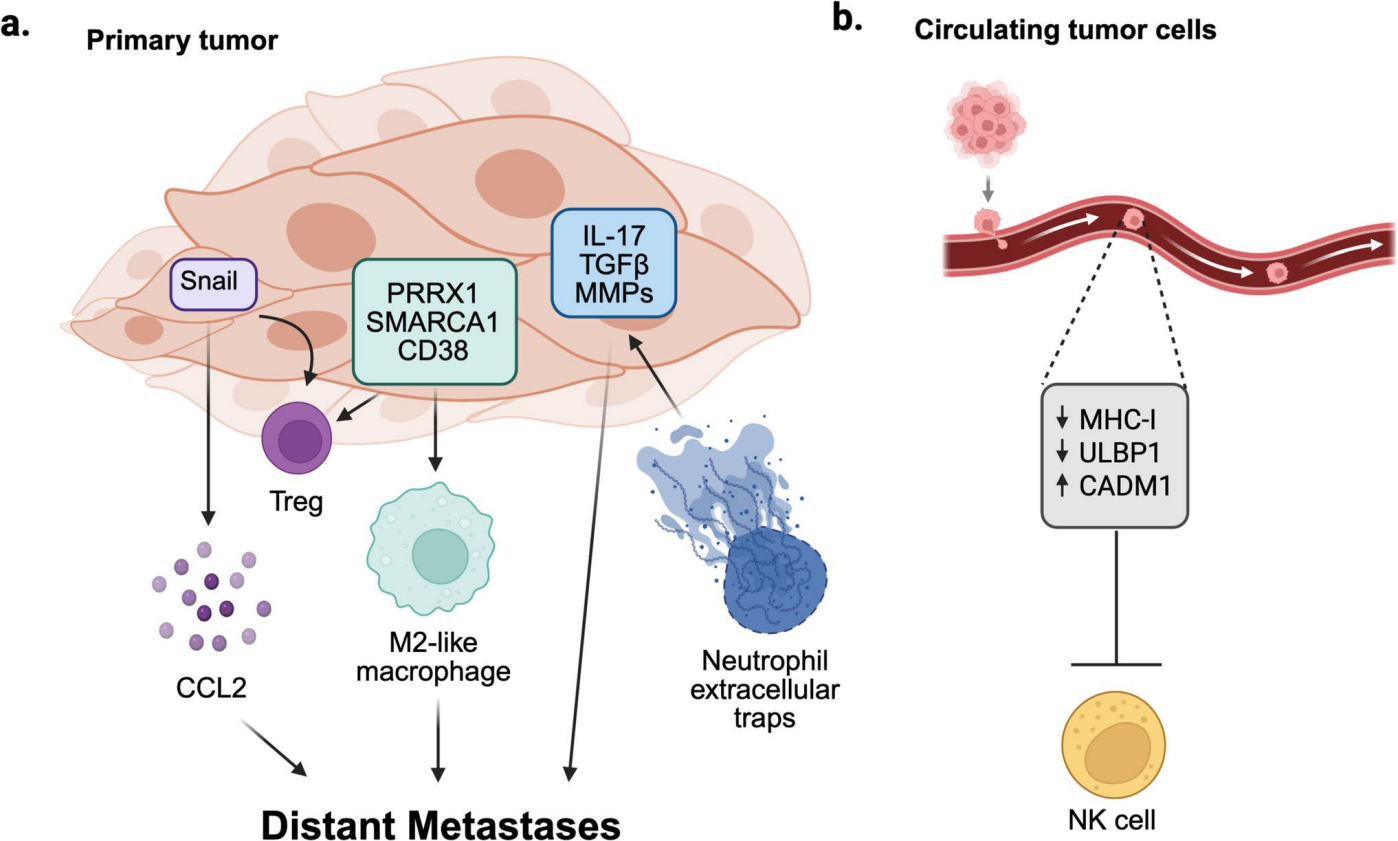
EMT-induced immunosuppression resulting in distant metastases. **a** Heterotypic interactions between mesenchymal cancer cells and immune cells within primary tumors facilitate distant metastasis. SNAIL-expressing cancer cells release CCL2 and induce the formation of Tregs which promote metastasis. Expression of the EMT-transcription factors PRRX1 and SNAIL, or CD38 and SMARCA1 results in an influx of M2-like macrophages into primary tumors resulting in metastasis. Neutrophil extracellular traps release cytokines and matrix metalloproteinases (MMPs) which activate an EMT in cancer cells leading to metastasis. **b** Circulating tumor cells which display features of an EMT program show altered expression of activating and inhibitory NK-ligands which shields them from NK cells in circulation, promoting metastasis

Metastatic colonization of distant tissues is often the leading cause of mortality for many different cancer types. Metastasis is a complex multi-step process, often referred to as the invasion-metastasis cascade collectively comprising of several distinct processes that eventually culminate in the forming of distant metastases [103–105]. These steps include the detachment of cancer cells from the primary tumor, intravasation, survival as solitary cells or clusters of circulating tumor cells in circulation and extravasation into distant organs [106–108]. At these foreign sites, surviving cancer cells either lie dormant for several years or alternatively, proliferate to form micro and subsequently, macrometastases [109, 110]. Indeed, the EMT program has been thought to regulate several steps of the invasion-metastasis cascade [103, 105]. In addition to activating the EMT program, cancer cells actively evade immunosurveillance at distinct stages of the invasion-metastasis cascade [105, 111]. In this section, we review (i) mechanisms of EMT-driven immunosurveillance at various steps of the invasion-metastasis cascade and; (ii) outline the mechanisms by which EMT-induced immunosuppression aids metastasis (Fig. 2).

Several studies have demonstrated that circulating tumor cells (CTCs) express markers associated with a hybrid or mesenchymal state and exhibit invasive features [112, 113]. One study demonstrated that CTCs from breast cancer patients exist as single or multicellular clusters and display elevated expression of mesenchymal markers such as TGFβ and the FOXC1 transcription factor [113]. Another study revealed that CTCs consist of clusters of cells expressing cytokeratin 14 (K14), which was found to be functionally essential for regulating metastasis [107]. As mentioned previously, mesenchymal cancer cells at the primary tumor site escape immunosurveillance by downregulating the expression of MHC-I. In analogous findings, altered expression of MHC genes has also been observed in CTCs that reside in a mesenchymal or hybrid state, demonstrating an association between activation of an EMT program and expression of cancer cell-intrinsic markers of immunosurveillance at both, the primary tumor site and post-intravasation [107, 114].

This altered expression of MHC molecules has been shown to promote immunoevasion by modulating the susceptibility of CTCs to NK cells. Susceptibility to NK cell-mediated lysis is governed by a balance between the loss of cell-surface MHC-I molecules and increased expression of activation ligands by target cells [115]. Malladi and colleagues observed that latency-competent cancer cells exhibit stem cell-like features and express the EMT-transcription factors SOX2 and SOX9. Inhibition of Wnt signaling leads to a reduction in the cell-surface expression of NK cell activating ligands such as ULBP1 [116]. This enables latency competent cells to evade clearance by NK cells and remain dormant [116]. In another study, human lung cancer cells that co-expressed epithelial and mesenchymal markers and resided in a hybrid state, had the highest propensity to metastasize relative to their epithelial or mesenchymal counterparts. Moreover, cancer initiating cells within this hybrid population had impaired secretion of chemo-attractants for NK cells and evaded NK cell mediated lysis by upregulating inhibitory ligands [117].

Working in the opposite direction, EMT-driven downregulation of MHC-I can also promote NK cell mediated lysis. Chockley and colleagues observed that the EMT program enhanced susceptibility to NK cell-mediated lysis by downregulating MHC-I and inducing the expression of CADM1, an NK cell activating ligand [118]. Intriguingly, deletion of NK cells promoted spontaneous metastasis, without affecting primary tumor growth [118]. Cheung and colleagues demonstrated that CTCs are comprised of epithelial-like clusters expressing K14. These clusters, which disseminate collectively to colonize distant organs, also lacked the expression of both, MHC-I and MHC-II genes, indicating cell-intrinsic mechanisms of immunoevasion [107]. Indeed, these K14 expressing cells were eliminated via NK cell mediated cytotoxicity in *ex vivo* co-culture assays [114].

In addition to the specific role of CTCs, several studies have also implicated that EMT-induced immunosuppression does, in fact, aid metastasis by employing adaptive immune cells. Expression of the EMT-TF SNAIL in melanoma cells leads to the induction of Tregs and the impairment of dendritic cell activity. Moreover, siRNA-mediated inhibition of SNAIL reverses EMT-induced immunosuppression leading to an impairment in the invasive capacity of these melanoma cells [19]. In subsequent work, the authors demonstrated that SNAIL-expressing melanoma cells secreted CCL2, which was critical for EMT-induced immunosuppression and metastasis [92]. Along similar lines, overexpression of the melanoma-associated antigen C3 (MAGE-C3) in esophageal squamous cell carcinoma cells led to the activation of an EMT program and the assembly of an immunosuppressive tumor microenvironment. Importantly, mice bearing MAGE-C3 overexpressing tumors had increased lung metastasis with increased numbers of dysfunctional CD8^+^ PD1^+^ T cells and lower numbers of total CD8^+^ T cells [119]. Metastatic breast tumor cells also release TβRII containing extracellular vesicles which are taken up by CD8^+^ T cells leading to sustained intracellular TGFβ signaling and exhaustion [120].

EMT-induced immunosuppressive effects on innate immune cells can also lead to subsequent metastasis. Downregulation of a lncRNA (LNMAS) in lymph node positive cervical cancer patients correlated with lymph node metastasis and prognosis [121]. LNMAS suppressed metastasis by interacting with HMGB1 thereby inhibiting chromatin accessibility by the EMT-TF TWIST. This, in turn, inhibited a TWIST-driven p-EMT and immunosurveillance by macrophages [121]. A recent study by Wang and colleagues demonstrated that SMARCA1 is a metastasis suppressor and inhibits transcription of NPFF by impairing the DNA binding capacity of its transcriptional regulator SPIB. Accordingly, SMARCA1-deficient colorectal cancer cells have increased NPFF secretion, which induces an M2-like state in TAMS resulting in the activation of an EMT. Thus, SMARCA1-NPFF axis is a dual regulator of EMT-driven metastasis and immunoevasion [122]. Additionally, a recent study demonstrated the EMT transcription factor PRRX1 drives an invasive trajectory in cancer cells that results in recruitment of pro-tumor macrophages and metastasis [123]. In another study by Cao et al., GPNMB expressed by cancer cells promoted polarization of monocytes to tumor-associated macrophages by establishing a feed-forward amplification loop. Importantly, dual inhibition of Siglec-E (murine Siglec-9 ortholog) and PD-1 reduced tumor stemness, suppressed EMT, and limited lung metastasis *in vivo*. The GPNMB-Siglec-9 axis thus represents a critical glycol-immunological checkpoint driving TAM-mediated metastasis, providing a promising therapeutic target in triple negative breast cancers [124]. Associations between EMT-induced immunosuppressive effects and metastasis have also been observed at the brain-tumor interface in meningiomas. A subset of metastatic cancer cells at this interface display a pro-EMT signature and co-localize with SPP1-expressing immunosuppressive macrophages, pericytes and endothelial cells [125].

In addition to macrophages, several studies have underscored the importance of neutrophils in driving EMT-induced immunosuppression resulting in metastatic colonization. This is largely due to the ability of a subset of neutrophils to form neutrophil extracellular traps (NETs) that activate an EMT program in adjacent cancer cells, enhancing their invasive and migratory capacities. Accordingly, blocking NETs can impair the activation of an EMT program and influence the pro-metastatic features of cancer cells [126–129]. In addition to NETs, neutrophils secrete several cytokines such as IL-17 and TGFB1 and remodel the extracellular matrix, all of which activate an EMT in cancer cells resulting in enhanced invasion, migration and metastatic ability [127, 130]. Finally, cross-talk between neutrophils with other components of the TME can also result in EMT-induced immunosuppression and metastasis. A recent study by Huang and colleagues showed that bile acids activate a G-protein coupled receptor on cancer associated fibroblasts inducing the release of CXCL10. This in turn results in the recruitment of neutrophils which assemble an immunosuppressive TME resulting in activation of an EMT program and metastasis in a model of cholangiocarcinoma [125].

Collectively, the aforementioned studies imply that heterotypic interactions between EMT-like cancer cells and immune cells results in immunosuppression that subsequently aids metastatic colonization. In addition to immune cells within the TME, targeting certain cancer-cell intrinsic factors that are specifically associated with an EMT-like state also impairs metastasis and potentiates the efficacy of immune checkpoint blockade therapies, at least in preclinical murine models. We have demonstrated that quasi-mesenchymal breast cancer cells express CD73 (an ectoenzyme that generates immunosuppressive adenosine) relative to their epithelial counterparts. Targeting CD73 not only reverses immunosuppression but also synergizes with anti-CTLA4 immune checkpoint blockade therapy to reduce lung metastasis [15]. Complementary findings by Visal and colleagues showed that breast cancer cells that reside in a hybrid state express CD38, another enzyme associated with the adenosinergic signaling pathway. These CD38-expressing hybrid EM cancer cells are highly invasive and motile and assemble an immunosuppressive TME. Targeting CD38 in combination with PD-L1 blockade reduces CTCs and lung metastases which is accompanied by a concomitant increase in CD8^+^ T cells and M1-like anti-tumor macrophages [78]. A recent study by Rozalen and colleagues demonstrated that the immune checkpoint receptor, TIM3, which is expressed by T cells, is also expressed by cancer cells. Strikingly, these TIM3-expressing cancer cells exhibit features of an EMT-program and are immunoevasive, leading to the formation of gamma-delta T cells and reducing CD8^+^ T cells during micrometastasis. Importantly, TIM3 blockade in the neoadjuvant setting mitigates metastatic seeding and initiation [131].

## Conclusions and future perspectives

Over the last decade or so, the EMT program has been increasingly recognized as a driver of immunosuppression and immunoevasion. Moreover, in addition to its well documented role in inducing stemness, driving metastatic colonization, and promoting resistance to chemo and targeted therapies, the EMT program has also been associated with resistance to certain forms of immunotherapy. While the mechanistic underpinnings of EMT-driven immunosuppression are beginning to emerge, it remains to be determined whether commonalities exist between EMT-associated drivers of resistance across multiple cancer types. For instance, components of the adenosinerigic signaling axis have been associated with driving resistance of quasi-mesenchymal breast cancer cells to anti-tumor immunity and immune checkpoint blockade therapies. However, whether the same signaling axis also drives immunotherapy resistance of other cancer types that have activated the EMT program is unknown. Indeed, lung cancer cells that have activated the EMT program express a distinct set of cancer cell-intrinsic markers as do certain pancreatic cancers and melanomas. Nevertheless, these diverse markers are all associated with immunosuppression and upregulated intrinsically within cancer cells that have activated the EMT program.

These findings give rise to the larger outstanding question of how do EMT-activated cancer cells express immunoevasive and immunosuppressive factors to begin with? While certain studies have suggested a role for canonical EMT-TFs in directly driving the expression of these factors by transcriptional regulation, whether other oncogenic signaling pathways collaboratively regulate the expression of immunoevasive features in hybrid or quasi-mesenchymal cancer cells requires further insight. Moreover, whether different EMT-TFs regulate distinct immunosuppressive trajectories of cancer cells is also unknown.

The EMT program has been shown to regulate the assembly of an immunosuppressive TME at the primary tumor site. Given the association of the EMT program in regulating metastatic colonization, whether EMT-induced immunosuppression is also observed during the invasion-metastatic cascade is an active area of investigation. While EMT-induced immunosuppression can certainly facilitate metastasis by shielding cancer cells in circulation from being eliminated by the immune system, there remain several outstanding questions. Firstly, whether the type of immune cells that congregate within the primary tumor site of EMT-activated tumors is the same or different from those present within distal metastases is unknown. Most cancer cells undergo the reverse process of mesenchymal-to-epithelial transition, or MET, in order to colonize distant tissues. Implicit in this phenomenon, is the notion that such a reversal may alter the identity of immune cells present within distal metastases. Additionally, how does organ tropism affect the composition of immune cells within the tumor microenvironment? One possibility is that each organ site could enrich for its own unique subset of immunosuppressive cells. Undertaking studies that longitudinally track how immune cells evolve after activation of the EMT program would shed insight into these questions.

Perhaps the most important aspect of associating the EMT program with immunosuppression, are the opportunities for intercepting this connection to potentiate the efficacy of immunotherapies. Immunotherapy in general and checkpoint inhibition in particular, have revolutionized cancer treatment. However, the success of these therapies has been blunted by the fact that only a subset of cancer types mount efficacious responses. Mesenchymal cancer cells represent a potent source of immunosuppression across multiple cancer types. Accordingly, designing strategies that could target EMT-like cancer cells holds the potential to be doubly beneficial because (i) it could eliminate a major source of immunosuppression within tumors that drives resistance to most standard of care immunotherapies (ii) Importantly, EMT-like cancer cells drive metastatic colonization, which is the cause of most cancer-associated mortalities. Thus, strategies to eliminate them altogether could have a dramatic impact on not only reversing immunosuppression within the TME but also inhibiting metastatic outgrowth. Taken together, such approaches could significantly inform rational combinatorial strategies to that could be transformative for cancer treatment.

## Data Availability

Not Applicable.
